# Band Gaps of Ho_*x*_Sc_1−*x*_N, Ho_*x*_Y_1−*x*_N, and Y_*x*_Sc_1−*x*_N Semiconductor Alloys

**DOI:** 10.3390/ma19194037

**Published:** 2026-09-22

**Authors:** Maciej J. Winiarski

**Affiliations:** Institute of Low Temperature and Structure Research, Polish Academy of Sciences, Okólna 2, 50-422 Wrocław, Poland; m.winiarski@intibs.pl

**Keywords:** nitride semiconductors, band gap, calculations

## Abstract

The electronic structure of ternary alloys of ScN, YN and HoN semiconductors has been studied with density functional theory calculations. The linear dependence of a band gap on an alloy content was predicted for these systems within the MBJGGA approach. The range of indirect band gaps $E_{g}^{\Gamma -X}$ available in Sc- and Y-based nitrides, starting from 0.89 and 1.09 eV, respectively, can be extended up to 1.3 eV thanks to alloying with HoN. The lack of band gap bowing found with the use of the special quasirandom structures for the $Y_{x}$${\mathrm{Sc}}_{1-x}$N systems is inconsistent with previous reports in the literature. The results presented in this work not only encourage further experimental efforts in band gap engineering in rare-earth nitrides, but also emphasize general requirements for theoretical studies on the electronic structure of this family of materials, which minimize the artificial band gap bowing found for the rock-salt nitride systems in previous studies.

## 1. Introduction

Semiconducting rare-earth (*RE*) nitride materials draw interest due to their promising applications in optoelectronics [1]. The recent advances in piezoelectric microelectromechanical ${\mathrm{Sc}}_{x}$${\mathrm{Al}}_{1-x}$N systems demonstrate the great potential of RE and group III nitride alloying in the search for novel functional materials [2,3,4,5,6,7,8,9,10]. Indirect band-gaps ($E_{g}^{\Gamma -X}$) in the range of 0.9–1.3 eV were reported for ScN, YN and LuN [11,12,13,14,15]. Unlike the hexagonal wurtzite group III nitrides (AlN, GaN, InN), *REN* compounds adopt the cubic rock-salt structure. Theoretical investigations and experimental studies on *REN* parent compounds and Sc-doped GaN systems have shown a tendency to form flat hexagonal (hBN-type) atomic layers in these materials under tensile strain [16,17,18,19,20]. The electronic structure of hexagonal *REN* monolayers was extensively studied in recent years [21,22,23,24,25,26,27,28]. The presence of strong spin–orbit coupling (SOC) in hexagonal LuN is connected with characteristic features of band structures similar to those in monolayers and heterostructures of transition metal dichalcogenides [29], which are considered a promising subject for research on valleytronics [30]. An exceptionally wide range of band gaps from 1.39 (bulk) up to 3.59 eV (three layers) was predicted for hexagonal multilayers of ScN [31]. The evolution of electronic structure in two-dimensional ScN systems is related to strong separation of orbital characters of valence bands, similar to that reported for the hexagonal multilayers of BN [32].

The open *f*-electron shells in lanthanide nitrides lead to robust ferromagnetic (FM) ordering, which is desired in spintronics and cryogenic electronics [33]. The early studies based on density functional theory (DFT+*U*) suggested relatively complex electronic structures of FM *REN* systems [34]. The indirect band gaps found for this family of compounds were relatively narrow, and some materials were predicted to be metallic due to the presence of 4f states in the vicinity of the Fermi level. The hybridization between the Ho 4*f* and N 2*p* states was further confirmed in experimental work [35]. An accurate description of electronic structure in the case of the *f*-electron *REN* compounds is a challenging task because of the simultaneous semiconducting and strongly correlated characters of the *p*-*d* and *f*-electron subsystems. According to the results of modern MBJLDA+*U* and dynamical mean-field theory (DMFT) calculations [36,37], the f-electron bands in lanthanide nitrides are localized and separated from the bands forming the valence band maximum (VBM) and conduction band minimum (CBM). The DMFT-derived width of the optical gap $E_{g}^{X-X}$ in this family of compounds is inversely proportional to the atomic number of a particular lanthanide element. These predictions are supported by the new experimental findings for the ${\mathrm{Ho}}^{3+}$ ions in high-quality thin films of HoN obtained with molecular beam epitaxy (MBE) [38]. Furthermore, the band gaps $E_{g}^{\Gamma -X}$ of DyN, HoN, and ErN are expected to be wider than the results of previous predictions based on the standard exchange–correlation functionals [34,37]. Unfortunately, except the extensively studied case of ScN, only reports on optical gaps $E_{g}^{X-X}$ in *RE* nitrides are available in the literature, because the measurements of indirect band gaps are difficult to perform. The research on the electronic structure of these materials requires further efforts in the synthesis of high-quality samples.

The range of available band gaps in cubic *REN* systems may be extended by formation of solid solutions and doping with group III elements [39,40]. The rock-salt solid solutions ${\mathrm{in}}_{x}RE_{1-x}$N may exhibit strongly increased $E_{g}^{\Gamma -X}$ with respect to those of the parent *REN* compounds, which is related to the chemical pressure caused by ${\mathrm{In}}^{3+}$ ions [39]. Similarly to the reports on the electronic structure of ternary alloys of group III nitrides [41], a pronounced band gap bowing was predicted for $Y_{x}$${\mathrm{Sc}}_{1-x}$N and ${\mathrm{Lu}}_{x}$${\mathrm{Sc}}_{1-x}$N materials. This effect was obtained for relatively homogeneous atomic configurations and is connected with the chemical pressure of bigger *RE* ions acting on the host ScN material. The strain-induced changes in $E_{g}^{\Gamma -X}$ in *REN* compounds are very rapid and may lead to metallic character in systems with strongly reduced unit cell volumes [18]. A smaller ionic radius mismatch between Y and Lu therefore leads to a smaller band gap bowing in ${\mathrm{Lu}}_{x}$$Y_{1-x}$N than that in $Y_{x}$${\mathrm{Sc}}_{1-x}$N, whereas the formation of ${\mathrm{La}}_{x}$${\mathrm{Sc}}_{1-x}$N solid solutions may result in metallic materials [40].

In this work, the electronic structure of ternary solid solutions of ScN, YN and HoN is studied with DFT-based methods. The origin of band gap bowings in *REN* materials is examined with the use of special quasirandom structures [42,43], which are periodic supercell approximations designed to correctly model the disordered alloys. The modified Becke–Johnson potential [44], designed for studies on the electronic structure of semiconducting materials, is used with the generalized gradient approximation (GGA and GGA+*U* [45]) in order to obtain reasonable predictions of structural parameters and band structures of the studied nitrides. The discussion is mainly focused on the dependence of band gaps on the content of ternary *REN* systems and the possible application of holmium in band gap engineering in ScN and YN semiconducting materials. High-quality Ho-doped thin films are expected to be feasible to grow with MBE, because this element exhibits only the 3+ valence state and easily reacts with molecular nitrogen. Furthermore, the comparable radius of ${\mathrm{Ho}}^{3+}$ and $Y^{3+}$ ions should lead to small lattice mismatch between the Ho- and Y-based systems, which is generally desirable for further experimental efforts in the synthesis of novel semiconducting materials based on rock-salt nitrides.

## 2. Materials and Methods

The 32-atom rock-salt special quasirandom structures were generated with the mcsqs package [43]. Taking into account the 16 transition metal sites available in the supercells, the smallest alloy content studied in this work is 0.0625 (6.25%). The DFT calculations were done with the VASP 5.4.4 package [46,47]. The Perdew–Burke–Ernzerhof (GGA [45]) exchange–correlation functional and the modified Becke–Johnson potential (MBJ [44]) were used. The full optimization of structural parameters of all systems was performed within the GGG/GGA+U approach, whereas the band structures were obtained with the MBJGGA calculations. The spin–orbit coupling was included. The plane-wave augmented (PAW [48]) pseudopotentials with semi-core states were employed. The f-electrons of the Ho ion were treated as valence states in the ferromagnetic phase and core states in the nonmagnetic phase. The Coulomb $U_{eff}$ parameter of 8.0 eV was applied to the Ho 4f states in the FM phase, following the previous studies on the electronic structure of HoN [34]. The valence basis sets were Sc: $3s^{2}3p^{6}3d^{2}4s^{1}$, Y: $3s^{2}4p^{6}5s^{2}4d^{1}$, ${\mathrm{Ho}}^{3+}$: $5p^{6}5d^{1}6s^{2}$, Ho: $4f^{10}5s^{2}5p^{6}5d^{1}6s^{2}$. The 400 eV plane-wave energy cutoff was set. The **k**-point meshes of 16 × 16 × 16 and 8 × 8 × 8 were selected for primitive and supercell systems.

## 3. Results and Discussion

The structural and electronic parameters calculated for the parent ScN, YN, and HoN compounds are gathered in Table 1. The GGA-derived lattice parameter *a* of ScN obtained here is in excellent agreement with the previous hybrid DFT (HSE06) and experimental results [49,50]. The similar findings for YN and HoN indicate a generally good description of the rock-salt ground state of *REN* nitrides within the GGA approach. The recent LDA calculations yielded underestimated unit cell volumes of (Sc; Y; La; Lu)N materials [18]. It is worth noting that the cubic lattice parameter of FM HoN obtained here with the GGA PAW pseudopotentials with the 4f-electron shell as valence states, as well as that obtained from the full-potential LDA calculations reported in the literature [37], is significantly underestimated with respect to the experimental one. It suggests that the structural parameters of Ho-bearing nitrides are more accurately modeled with the *f*-electrons treated as core states.

The band structures obtained with the MBJGGA calculations are depicted in Figure 1. The characteristic overall shapes of valence and conduction bands in *RE* nitrides, as well as the valence bandwidth, are connected to some extent to the ionic radii of particular *RE* elements. Similar band structures for ScN and YN were recently reported in the full-potential studies [18,40]. The Sc-based system exhibits a relatively narrow fundamental gap ($E_{g}^{\Gamma -X}$) and wide optical gap ($E_{g}^{X-X}$). The former increases in YN and HoN, whereas the latter is reduced in these systems. The fundamental and optical gaps in HoN differ only by 0.2 eV, which is further decreased in the FM phase. Such a small difference between $E_{g}^{\Gamma -X}$ and $E_{g}^{X-X}$ in *REN* materials may be difficult to distinguish in measurements. It is worth recalling that the exceptionally big ionic radius of La leads to the direct fundamental gap $E_{g}^{X-X}$ of 0.59 eV and a significant shift of valence bands at the Γ point in the Brillouin zone of LaN into the binding energy region well below VBM [18,40]. A similar band structure with the direct band gap $E_{g}^{X-X}$ was also obtained with the DMFT calculations for neodymium nitride [36]. One may consider that the alloying of ScN and YN with lanthanide nitrides based on bigger ions than ${\mathrm{Ho}}^{3+}$ may lead to complex evolution of electronic structure in such mixed systems.

As shown in Table 1, the MBJGGA calculations yield a very accurate value of $E_{g}^{\Gamma -X}$ in ScN compared to the previous hybrid DFT (HSE06) results and the experimental report on high-quality MBE-grown samples [49]. However, the MBJGGA-derived $E_{g}^{X-X}$ for this system is clearly underestimated. This effect may be explained to some extent by the Burstein–Moss shift, but the result of HSE06 calculations is very close to the measured one [49]. Although the experimental data for high-quality YN materials is not available, the modern DFT-based calculations predict $E_{g}^{\Gamma -X}$ and $E_{g}^{X-X}$ of 1.0–1.1 and 1.4–1.5 eV for this compound [18,40,51]. The optical gap obtained here for HoN is slightly narrower than the results of measurements for high-quality thin films. The values of $E_{g}^{\Gamma -X}$ for nonmagnetic and FM phases of HoN are very close to each other, which supports the general assumption of independent *p*-*d* and *f* electron subsystems in HoN. As shown in Figure 1c,d, the flat 4f bands are localized below the valence region of this compound. The effect of spin–orbit coupling (SOC) on the band structure predicted for HoN is clearly pronounced. The valence bands at the high-symmetry points in the Brillouin zone, almost degenerate in ScN (see Figure 1a), are strongly split in HoN. The influence of magnetic ordering on valence bands in this system is weaker than the SOC-driven effects. In view of the aim of this work, the nonmagnetic band structure is adopted as a reasonable model of the electronic structure of HoN.

The total and partial density of states (DOS) plots are presented in Figure 2. Despite the valence region in *REN* nitrides being dominated by the N 2p states, similar to the case of group III nitrides with fully occupied d-type orbitals, in the case of ${\mathrm{RE}}^{3+}$ ions in *RE* nitrides, their *d*-type contributions to the valence bands are significant and become completely dominant in the characteristic nitride-like tail of the conduction band region. The main peak of valence DOS in ScN exhibits a smaller width and is located at a higher binding energy than those in YN and HoN. The strongly reduced valence bandwidths and similar to each other overall shapes of the valence DOS are characteristic of these compounds. The slightly smaller valence bandwidth in HoN reflects the reduction in ionic radius between Ho and Y. One may expect similar effects in DyN and ErN, taking into account the ionic radii of ${\mathrm{Dy}}^{3+}$ and ${\mathrm{Er}}^{3+}$, which are comparable with that of ${\mathrm{Ho}}^{3+}$. The pronounced splitting of valence bands in HoN, related to strong SOC (see Figure 1c), is reflected in a flattened and less smooth DOS in this system.

The dependence of the cubic lattice parameter *a* on the content of the alloys studied is depicted in Figure 3. The results of theoretical structural optimization exhibit linear Vegard’s law behavior. The alloying of ScN with HoN and YN leads to significantly increased *a* in the resulting ternary materials, as shown in Figure 3a,c, whereas the variation in *a* in ${\mathrm{Ho}}_{x}$$Y_{1-x}$N, depicted in Figure 3b, is almost negligible. Bearing in mind the lack of suitable substrates, which is still a crucial issue for experimental studies on *REN* thin films [38], the equilibrium values of *a* obtained here for the parent ScN, YN, and HoN compounds (see Table 1) indicate that these materials are potential substrates for the growth of thin films of ternary alloys with reasonable lattice mismatch. The comparable value of *a* was also reported for LuN and other *f*-electron *RE* nitrides [34,37].

As presented in Figure 4a,b, the strong increase in band gaps ($E_{g}^{\Gamma -X}$) with an increasing holmium content is expected in the ${\mathrm{Ho}}_{x}$${\mathrm{Sc}}_{1-x}$N and ${\mathrm{Ho}}_{x}$$Y_{1-x}$N systems. No band gap bowing is found, i.e., the increase in $E_{g}^{\Gamma -X}$ is generally linear with some outliers for particular alloy contents. These effects are connected with the use of 32-atom SQS supercells, which are characterized by relatively complex and irregular shapes. The linear $E_{g}^{\Gamma -X}\left(x\right)$ was also found for solid solutions of *REN* and group III nitrides with *x* suitable for the stable rock-salt ground state [20]. Interestingly, the widest band gaps were obtained for ${\mathrm{In}}_{x}$${\mathrm{Sc}}_{1-x}$N, which is connected with chemical pressure and variation in valence electron configuration of ${\mathrm{Sc}}^{3+}$ and ${\mathrm{In}}^{3+}$ ions. The effect of doping with Ho or Y ions in the ScN host semiconductor is less complex, which may be desirable for feasible growth of ternary *RE* nitride systems with well-defined electronic structure.

It is worth noting that the dependence of $E_{g}^{\Gamma -X}$ in $Y_{x}$${\mathrm{Sc}}_{1-x}$N, presented in Figure 4c, does not support the findings of previous DFT-based calculations [40], which lead to a significant band gap bowing. The corresponding data taken from Ref. [40] suggest a lower $E_{g}^{\Gamma -X}$ with respect to that of the parent ScN compound in systems with x below 0.75. This issue is related to the periodic nature of supercell calculations, which can be diminished with the use of SQS systems even in the case of relatively large variation in ionic radii of particular elements in an alloy. One may expect that the high-quality samples should exhibit a rather linear dependence of $E_{g}^{\Gamma -X}$ on an alloy content. It is also worth recalling that the strong experimental band gap bowing in ${\mathrm{In}}_{x}$${\mathrm{Ga}}_{1-x}$N and ${\mathrm{In}}_{x}$${\mathrm{Al}}_{1-x}$N materials were explained based on the clustering of In ions. The results of supercell calculations with possibly homogeneous atomic configurations revealed a significantly smaller deviation from the linear Vegard’s-like behavior of band gaps in these systems. However, the band gap bowing in ${\mathrm{In}}_{x}$${\mathrm{Ga}}_{1-x}$N for low In contents was also reported in further SQS-based studies [53], which suggests that the local influence of In ions on the electronic structure of the host wurtzite GaN material is very strong. In the case of *RE* nitrides investigated in this work, such a phenomenon is not revealed because of the presence of unoccupied *d*-type states in *RE* ions and the rock-salt unit cell with a small number of structural parameters that can be locally relaxed.

As presented in the density of states (DOS) plots in Figure 5, the evolution of electronic structure in ${\mathrm{Ho}}_{x}$${\mathrm{Sc}}_{1-x}$N alloys is proportional to the Ho content. The overall shape of the total DOS in ${\mathrm{Ho}}_{0.25}$${\mathrm{Sc}}_{0.75}$N, shown in Figure 5a, resembles that of ScN (see Figure 1a). Although the Sc 3d contributions are significant in the valence band region and dominant above the band gap of this system, the amplitude of the main DOS peak, dominated by the N 2p states, is strongly reduced compared to that of the host ScN semiconductor. The Ho 5d contributions are minor, and their shape is similar to that of the Sc 3d states. They become stronger in ${\mathrm{Ho}}_{0.5}$${\mathrm{Sc}}_{0.5}$N, in which the *d*-type contributions from Sc and Ho ions are comparable, and dominate in ${\mathrm{Ho}}_{0.75}$${\mathrm{Sc}}_{0.25}$N. However, the characteristic asymmetry of the valence region connected with the Sc 3d states is still clear. Analogous effects are predicted for ${\mathrm{Ho}}_{x}$$Y_{1-x}$N but are less pronounced because of the great similarity between the overall shapes of the valence DOS regions in the parent YN and HoN compounds (see Figure 1b,c).

## 4. Conclusions

The electronic structure of ternary alloys of ScN, YN and HoN was investigated with DFT-based calculations. The band gaps obtained for ${\mathrm{Ho}}_{x}$${\mathrm{Sc}}_{1-x}$N and ${\mathrm{Ho}}_{x}$$Y_{1-x}$N alloys with the MBJGGA calculations demonstrate the potential role of holmium in band gap engineering in ScN and YN semiconductors. The use of this exchange–correlation functional is crucial because the standard DFT-derived band gaps are strongly underestimated. The predictions based on the MBJGGA approach are expected to accurately describe the electronic structure of semiconducting systems. The linear dependence of $E_{g}^{\Gamma -X}$ on an alloy content was found for the ternary materials, including $Y_{x}$${\mathrm{Sc}}_{1-x}$N, with a relatively large variation in ionic radii between scandium and yttrium. The use of SQS instead of simple supercells, which are common in theoretical studies, is crucial for the reduction of artificial band gap bowings in ternary solid solutions of *RE* nitrides. The SQS approach is designed to mimic properties of perfectly random alloys thanks to the carefully selected shapes of supercells, which minimize the errors related to the periodic nature of the plane-wave DFT calculations. The findings presented here question very strong band gap bowings in *REN* alloys reported in the literature and define new technical requirements for meaningful predictions of electronic structure for novel semiconducting nitride alloys.

## Figures and Tables

**Figure 1:**
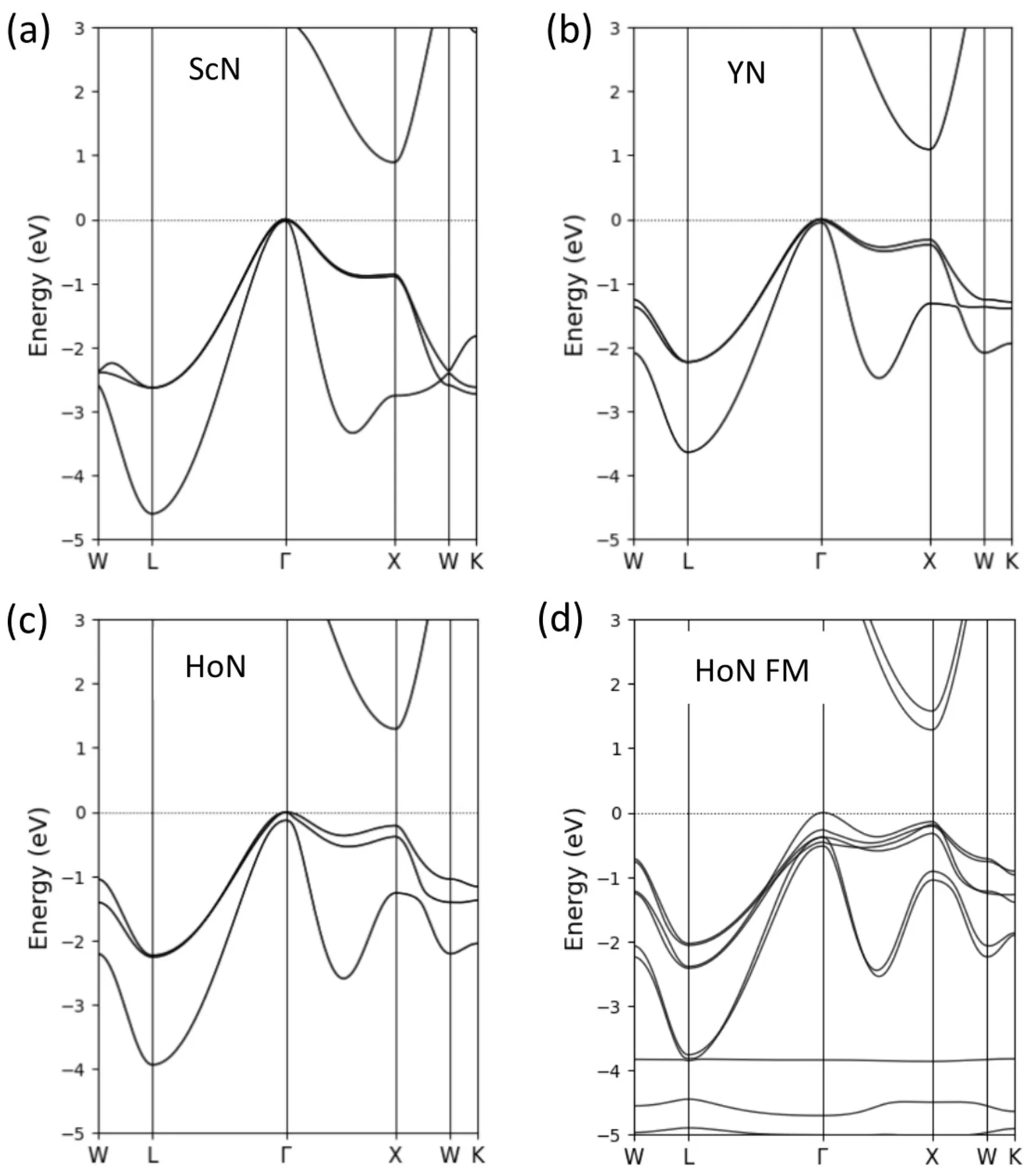
Band structures calculated within the MBJGGA approach for ScN (**a**), YN (**b**), and HoN in nonmagnetic (**c**) and ferromagnetic (**d**) phase.

**Figure 2:**
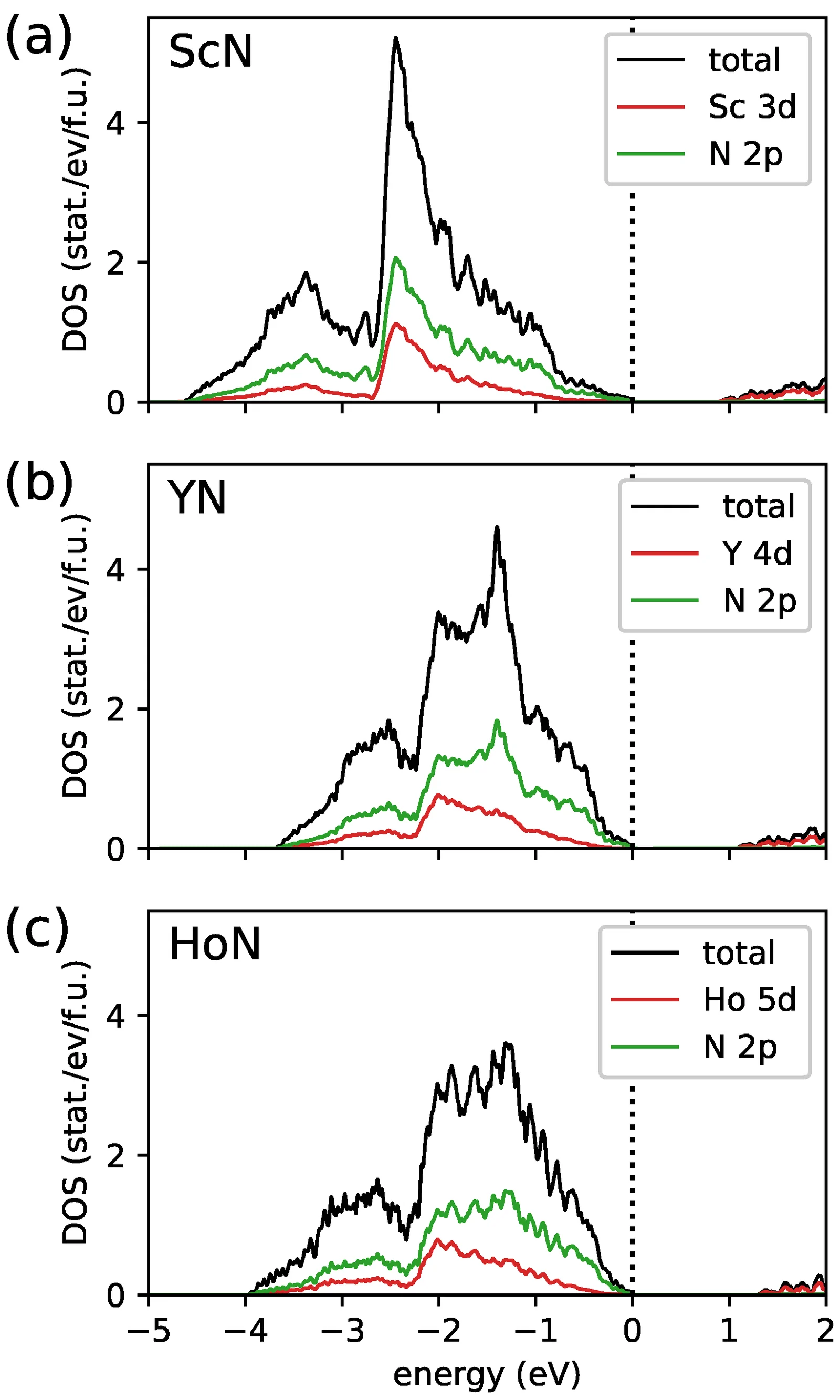
Total and partial density of states (DOS) calculated for ScN (**a**), YN (**b**), and HoN (**c**) parent compounds.

**Figure 3:**
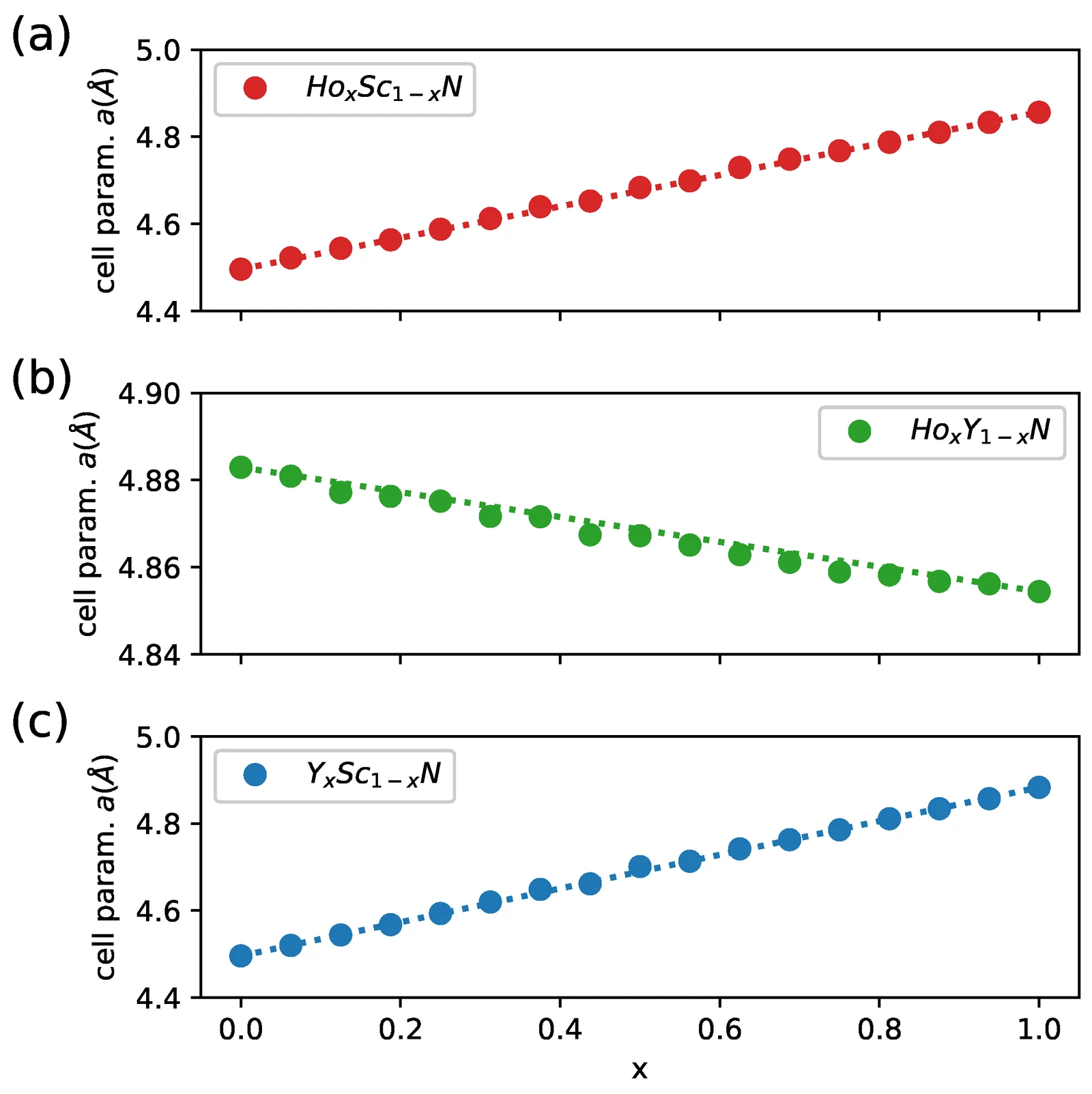
Cubic lattice parameter in ${\mathrm{Ho}}_{x}$${\mathrm{Sc}}_{1-x}$N (**a**), ${\mathrm{Ho}}_{x}$$Y_{1-x}$N (**b**), and $Y_{x}$${\mathrm{Sc}}_{1-x}$N (**c**) alloys. Vegard’s law is marked with dashed lines.

**Figure 4:**
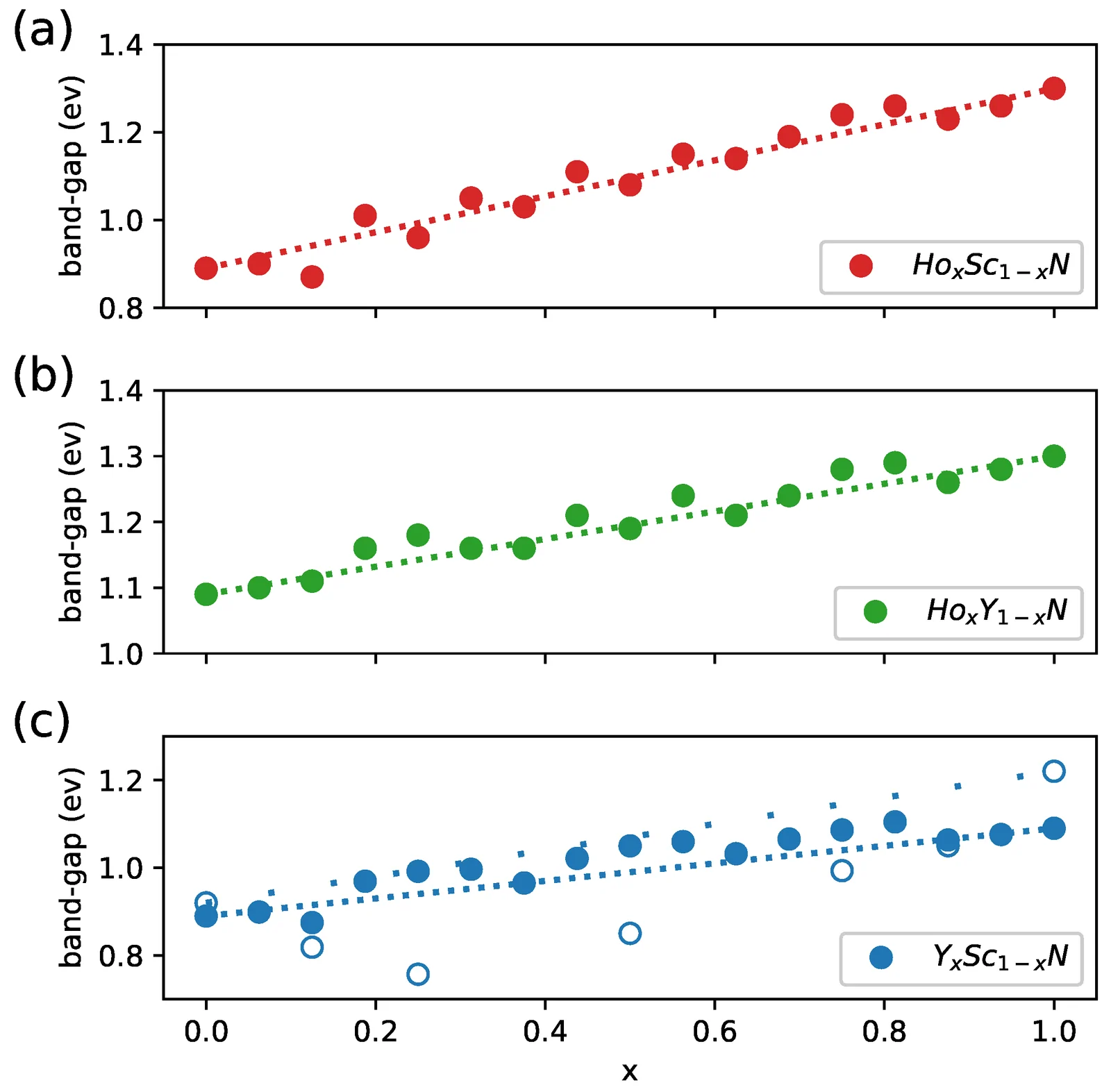
Band gaps calculated within the MBJGGA approach for ${\mathrm{Ho}}_{x}$${\mathrm{Sc}}_{1-x}$N (**a**), ${\mathrm{Ho}}_{x}$$Y_{1-x}$N (**b**), and $Y_{x}$${\mathrm{Sc}}_{1-x}$N (**c**) alloys. Vegard’s law is marked with dashed lines. The reference data for $Y_{x}$${\mathrm{Sc}}_{1-x}$N taken from Ref. [40] are marked with open circles and a dotted line.

**Figure 5:**
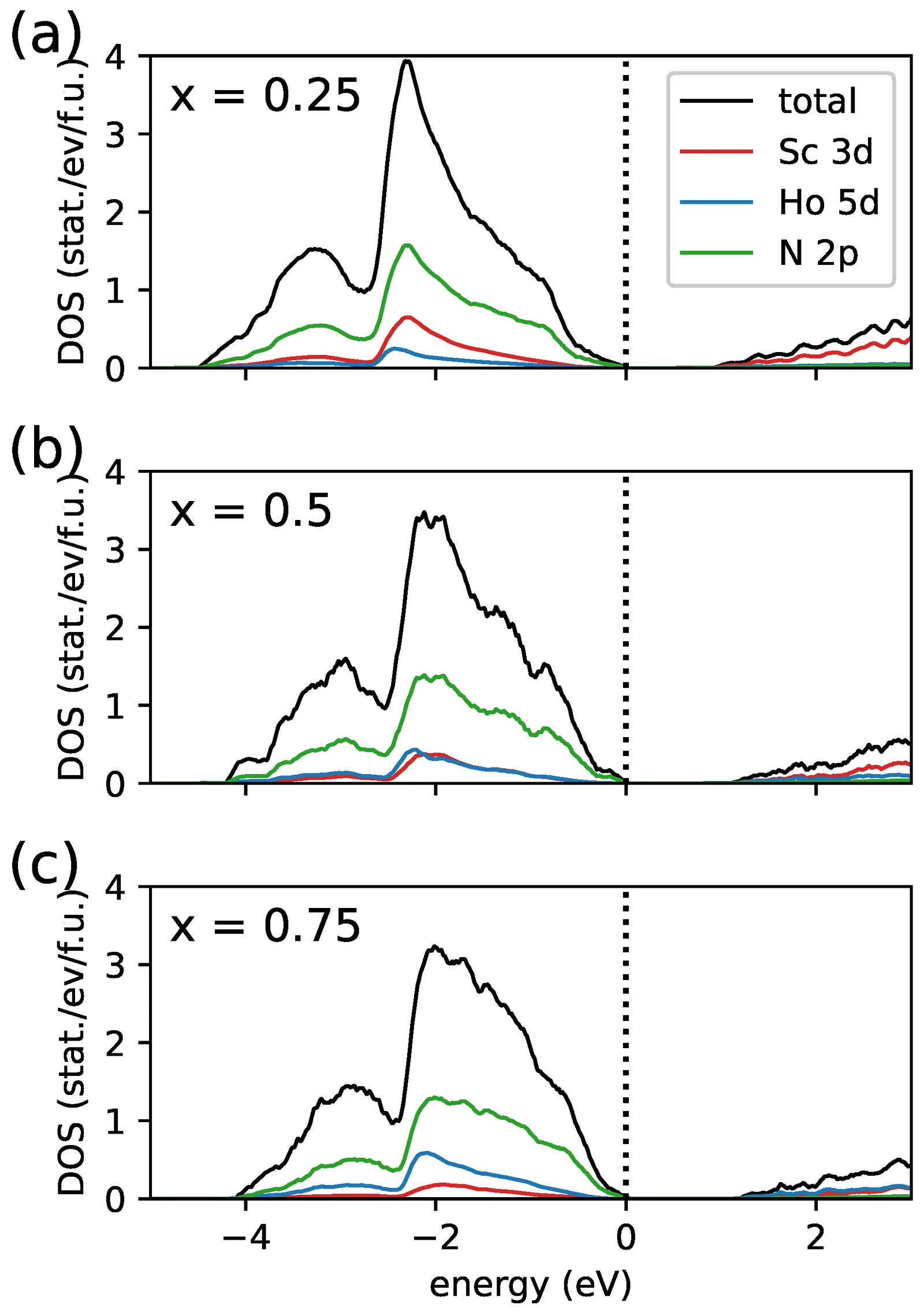
Total and partial density of states (DOS) calculated for ${\mathrm{Ho}}_{x}$${\mathrm{Sc}}_{1-x}$N alloys with x = 0.25 (**a**), 0.5 (**b**), and 0.75 (**c**).

**Table 1:** The cubic lattice parameters *a*, fundamental and optical gaps calculated in this work, and the available literature data for ScN, YN, and HoN.

System	*a* (Å)	$E_{g}^{\Gamma -X}$ (eV)	$E_{g}^{X-X}$ (eV)
ScN:			
this work	4.4959	0.89	1.74
Ref. [49]	4.499	0.92	2.02
Experim.	4.501 [50]	0.92 [49]	2.07 [49]
YN:			
this work	4.8829	1.09	1.41
Ref. [51]	4.93	0.97	1.46
Experim.	4.877 [52]	-	-
HoN:			
this work	4.8562	1.30	1.51
Experim. [35]	4.871	-	1.48
FM HoN:			
this work	4.7836	1.28	1.43
Ref. [37]	4.7270	0.97	1.17
Ref. [34]	-	0.48	1.00

## Data Availability

The original contributions presented in this study are included in the article. Further inquiries can be directed to the corresponding author.
